# Targeted Therapies and Immune Checkpoint Inhibitors in Primary CNS Lymphoma

**DOI:** 10.3390/cancers13123073

**Published:** 2021-06-20

**Authors:** Hans-Georg Wirsching, Michael Weller, Stefan Balabanov, Patrick Roth

**Affiliations:** 1 Department of Neurology and Brain Tumor Center, University Hospital Zurich, 8091 Zurich, Switzerland; hans-georg.wirsching@usz.ch (H.-G.W.); michael.weller@usz.ch (M.W.); 2 Department of Medical Oncology and Hematology, University of Zurich, 8091 Zurich, Switzerland; stefan.balabanov@usz.ch

**Keywords:** primary CNS lymphoma, PCNSL, ibrutinib, lenalidomide, immune checkpoint inhibition, PD-1, nivolumab, pembrolizumab

## Abstract

**Simple Summary:**

Primary central nervous system lymphoma is a rare disease with limited therapeutic options. A more profound understanding of the molecular mechanisms that underlie this disease has fostered the development of novel therapeutic approaches. Key developments for the treatment of primary central nervous system lymphoma were made in the field of small molecule inhibitors, i.e., drugs that were designed to specifically target the molecular backbone of cancer. Prominent examples include inhibitors of Bruton’s tyrosine kinase or mammalian target of rapamycin, as well as immune modulatory thalidomide analogues. Along the same lines, another major strain of drug development for primary central nervous system lymphoma comprises of immune checkpoint inhibitors, i.e., monoclonal antibodies designed to unleash anti-cancer immune reactions. This review article discusses these ongoing clinical developments, including biological rationale as well as preliminary toxicity and efficacy, and provides an outlook for future developments.

**Abstract:**

This review article outlines the current development of emerging treatment strategies for primary central nervous system lymphoma, a rare brain tumor with, thus far, limited therapeutic options. Small molecule targeted tyrosine kinase inhibitors, immunomodulatory agents, and immune checkpoint inhibitors will be discussed. The mechanisms of action, results of completed clinical studies, ongoing clinical trials, and future perspectives are summarized. Among the most promising clinical developments in the field of CNS lymphomas is ibrutinib, an inhibitor of Bruton’s tyrosine kinase, which relays activation of nuclear factor kappa B upon integration of constitutive B cell receptor and Toll-like receptor signals. Down-stream of nuclear factor kappa B, the thalidomide analogs lenalidomide and pomalidomide exert immunomodulatory functions and are currently explored against CNS lymphomas. Finally, immune checkpoint inhibitors, such as drugs targeting the PD-1 pathway, may become novel therapeutic options to unleash anti-tumor immunity in patients with primary CNS lymphoma.

## 1. Background

Primary central nervous system lymphoma (PCNSL) is an extra-nodal lymphoma characterized by exclusive localization in the central nervous system, including the leptomeningeal compartment and eyes. PCNSL is rare, accounting for approximately 2% of all primary brain tumors and less than 1% of non-Hodgkin lymphomas [1]. The incidence of PCNSL increases with age for unknown reasons, with a median age of 67 years at the time of diagnosis in the U.S. [1]. The histopathological assessment of the tumor tissue reveals a diffuse large B cell lymphoma (DLBCL) in the vast majority of cases [2].

Most standard regimens used for the treatment of patients with systemic lymphoma, such as R-CHOP, were proven inactive against PCNSL, most likely because of the very limited blood-brain barrier penetration of these drugs. Whole brain radiotherapy (WBRT) results in high response rates, but rarely in durable remissions. Furthermore, WBRT is associated with significant neurotoxicity, particularly in the overall elderly PCNSL population [3]. It has therefore been largely abandoned as part of first-line therapy and is now more frequently used as a salvage therapy in patients lacking other treatment options, with limited activity. High-dose intravenous methotrexate (HD-MTX) has become the backbone of virtually all treatment regimens for PCNSL as it crosses the blood-brain barrier and exerts clinically meaningful anti-lymphoma activity. In the last 20 years, most efforts aimed at improving the outcome of PCNSL patients involves combining HD-MTX with additional drugs, mostly classical chemotherapeutic agents. Among the drugs which have been used most frequently are high-dose cytarabine, ifosfamide, thiotepa, procarbazine, and vincristine. While these combinations have resulted in increased response rates, their impact on overall survival is less clear, partially because of the limited sample size of the respective trials [4,5,6]. WBRT and autologous stem cell transplantation were explored as a consolidation therapy. WBRT should be avoided, particularly in patients achieving a complete response, because of its negative impact on cognitive function [7,8]. Stem cell support may be overall better tolerated and, therefore, be the preferred consolidation strategy in patients qualifying for such an intense therapy [9,10].

Advances in the understanding of molecular mechanisms which drive PCNSL have triggered molecularly targeted treatment approaches. The first targeted drug that was frequently added to HD-MTX-based regimens was rituximab, a monoclonal antibody targeting CD20. Rituximab was approved for different types of systemic lymphomas with proven efficacy. While CD20 is expressed on largely all PCNSL, the value of rituximab against this tumor remains a matter of debate. Due to its overall good tolerability and the preliminary data from retrospective series which suggested activity against PCNSL, it was more frequently integrated into treatment protocols for PCNSL [11]. Rituximab was also added to HD-MTX and cytarabine in the induction part of the IELSG-32 trial. This approach translated into numerically higher response rates, as well as longer progression-free (PFS) and overall survival (OS) compared to chemotherapy alone [12]. These findings are in contrast to an adequately sized, randomized trial where rituximab was added to HD-MTX, procarbazine, and carmustine. In this study, there was a non-significant trend for longer PFS in the rituximab group, but no difference in OS between the two treatment arms [13]. Therefore, the role of rituximab in the treatment of PCNSL requires further research. A more detailed and critical evaluation on the role of rituximab is beyond the scope of this review and can be found elsewhere [14].

Within the past decade, several drugs have become available for compassionate use in PCNSL patients based on a continuously improving understanding of the molecular basis of the disease. Some of these agents have entered advanced-stage clinical development and may become part of the standard treatment of PCNSL in the future. Since the treatment of elderly PCNSL patients who are not eligible for an HD-MTX-based regimen remains a particular challenge [15], novel therapeutic strategies to improve the prognosis of this continuously increasing patient population are urgently needed. The following sections provide an overview on the rationale and current clinical development of novel drugs, which may display anti-tumor activity as single agents or as part of combination regimens with established chemotherapeutic agents, including small molecule inhibitors of key oncogenic pathways that underlie PCNSL and immune checkpoint inhibitors designed to unleash an anti-PCNSL immune response.

## 2. Targeted Therapies

**Figure 1:**
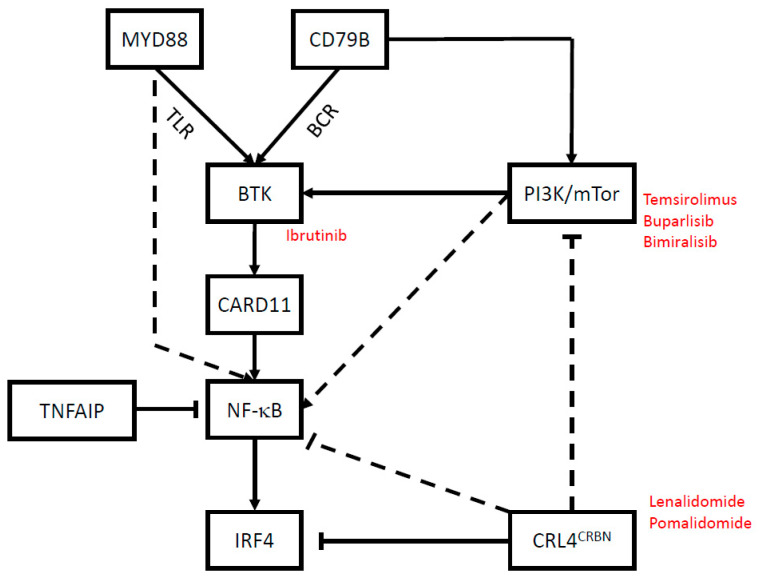
Schematic of oncogenic drivers of PCNSL. Small molecule inhibitors that are currently under clinical investigation for PCNSL are highlighted in red. Abbreviations: MYD88, myeloid differentiation primary response 88; BTK, Bruton’s tyrosine kinase; CARD11, caspase recruitment domain-containing protein 11; TNFAIP, tumor necrosis factor (TNF)-α-induced protein; NF-κB, nuclear factor kappa-light-chain-enhancer of activated B cells; IRF4, interferon regulatory factor 4; CRL4, cullin-RING E3 ubiquitin ligase 4; CRBN, cereblon; PI3K, phosphoinositide 3-kinase; mTOR, mechanistic target of rapamycin.

Different molecular subgroups of DLBCL were defined based on gene expression profiling and were termed: (i) germinal center B-cell-like (GCB), (ii) activated B-cell-like (ABC), and (iii) the poorly defined type 3 [16,17,18]. The oncogenic hallmark of PCNSL is the activation of the NF-κB (nuclear factor kappa-light-chain-enhancer of activated B cells), driven by constitutive activation of signaling through the B cell receptor (BCR) and Toll-like receptor (TLR) [19]. PCNSL harbor BCR-activating mutations in *CD79B* and TLR-activating mutations in *MYD88* at a higher frequency than systemic ABC type DLBCL, whereas oncogenic mutations in *CARD11* and *TNFAIP3* occur less frequently in PCNSL [20,21,22,23,24]. *MYD88* and *CD79B* mutations are absent in rare cases of immunosuppression-associated PCNSL, which are generally driven by the infection of B cells with Epstein-Barr virus [25]. In contrast to systemic DLBCL, *MYD88* and *CD79B* mutations also occur in non-ABC type PCNSL [21,26]. The frequent loss of chromosome 6q in PCNSL, where the NF-κB inhibitor *TNFAIP3* is encoded, may further contribute to NF-κB activation [27,28,29,30]. The BCR/TLR-NF-κB axis can be targeted at multiple levels: up-stream of NF-κB utilizing small molecule tyrosine kinase inhibitors, and down-stream of NF-κB utilizing immune-modulatory drugs (Figure 1).

### 2.1. Bruton’s Tyrosine Kinase: Ibrutinib

BCR and TLR signaling converge on the Bruton’s tyrosine kinase (BTK), which integrates both of the signals for subsequent down-stream activation of NF-κB [31]. BTK was identified as a prime molecular target in PCNSL and the BTK inhibitor ibrutinib has entered clinical development. In a phase 1/2 study in patients with recurrent or refractory PCNSL, a maximum tolerated dose of 840 mg daily oral, single-agent ibrutinib was determined and a clinical response was noted in 10 of 13 patients, including five complete radiographic responses, yielding a median progression-free survival of 4.6 months [21]. There was a single patient without clinical response, which was attributed to a *CARD11* mutation, a known mechanism of resistance to ibrutinib [21]. Moreover, *CD79B* mutations appeared to convey upregulation of phosphoinositide 3 kinase/mammalian target of rapamycin (PI3K/mTor) signaling as a potential resistance mechanism [21]. Sequential therapy of ibrutinib and HD-MTX was also explored in a separate arm of this trial and yielded clinical responses in 12 of 15 patients (80%), and durable response was associated with the clearance of circulating tumor DNA from the cerebrospinal fluid [32].

A phase 2 study exploring the tolerability and efficacy of 560 mg daily oral ibrutinib in 52 patients with PCNSL or ocular lymphoma in the recurrent setting found a median PFS of 4.8 months [33]. Imaging response was assessed in 42 patients after 2 months of study treatment and it identified 10 complete responses (19%) and 17 partial responses (33%), independently of mutations in *MYD88* or *CD79B* [33]. A third study explored the administration of ibrutinib during a 2-week window of opportunity in 18 patients with newly diagnosed or recurrent PCNSL, prior to a variety of different chemotherapy regimens [34]. Although high response rates were reported from this study, weighing the contribution of ibrutinib is challenging due to the variable subsequent treatments applied to patients [34].

Overall, ibrutinib was tolerated as a single agent with manageable toxicity that was resolved upon cessation of treatment, with the most common adverse events being neutropenia, lymphopenia, and three cases of fungal infection among 62 ibrutinib-treated patients with PCNSL, including one with a fatal outcome [21,33]. By contrast, around 40% of fungal infections were reported with a combination approach of ibrutinib with different chemotherapy regimens [34].

There are several ongoing early phase clinical trials of ibrutinib in combination with standard and experimental drugs in recurrent or refractory PCNSL (Table 1), including a randomized phase 2 trial comparing the addition of ibrutinib versus lenalidomide in combination with MTX, rituximab, and etoposide (NCT04129710). Furthermore, a phase 2 study explores ibrutinib in the newly diagnosed setting as a maintenance therapy in elderly patients (age 60–85) following induction treatment with HD-MTX plus rituximab (NCT02623010). The toxicity and preliminary efficacy of the second generation oral BTK inhibitor acalabrutinib is explored in a phase 1 study, in combination with the immune checkpoint inhibitor durvalumab, in patients with recurrent or refractory PCNSL (NCT04462328).

Of note, even though prolonged remissions during treatment with ibrutinib alone are seen in other lymphoid malignancies, the disease usually recurs promptly after stopping ibrutinib. Therefore, ibrutinib alone may not be curative, but rather support the efficacy of combination therapy regimens. 

### 2.2. PI3K/mTor: Temsirolimus, Buparlisib and Bimiralisib

Temsirolimus is an intravenously administered small molecule inhibitor of mTOR and was explored as single-agent therapy at up to 75 mg weekly in a phase 2 study that enrolled 37 patients with recurrent or refractory PCNSL [35]. The median age at enrollment was 70 years and the general condition of patients was good. Temsirolimus yielded an overall response rate of 54%, including five complete responses and 12 partial responses. However, responses were usually of short duration, resulting in a median PFS of only 2.1 months at the cost of considerable toxicity, with the most common adverse events being hyperglycemia, thrombocytopenia, infection, or anemia. This study demonstrated that mTor inhibition has biological activity in PCNSL, although resistance appears to evolve rapidly. The low cerebral spinal fluid concentrations of temsirolimus in this study suggest poor blood-brain barrier penetration and the dependence of efficacy on blood-brain barrier disruption. Further exploration of mTor as a molecular target in PCNSL for combination treatment approaches, particularly as a means to overcome resistance to ibrutinib in *CD79B*-mutant tumors, may be warranted. 

Buparlisib acts up-stream of mTor by inhibition of PI3K and was administered to four patients with PCNSL, yielding only a single partial response, likely due to limited penetration of the blood-brain barrier [36]. The buparlisib derivative bimiralisib (PQR309) is an oral, small molecule inhibitor of both PI3K and mTor that was designed to overcome this limitation and readily crosses the blood-brain barrier [37,38]. Pre-clinically, the activation of BCR signaling was associated with anti-lymphoma activity of bimiralisib and activity against lymphoma cell lines was observed in combination with other targeted therapies, including rituximab, ibrutinib, lenalidomide, ARV-825, marizomib, venetoclax, and panobinostat [39]. Bimiralisib obtained orphan drug status by the U.S. Food and Drug Administration and by the European Medicines Agency for the treatment of PCNSL. Publication of the results of a phase 2 study of daily oral bimiralisib, that accrued 21 patients with recurrent or refractory PCNSL, is expected in 2021 (NCT02669511). An ongoing phase 1/2 trial explores the combination of the PI3K inhibitor copanlisib with ibrutinib in patients with recurrent or refractory PCNSL (NCT03581942).

### 2.3. Immune Modulatory Small Molecules: Lenalidomide and Pomalidomide 

Lenalidomide and pomalidomide are more potent and less toxic brain-penetrating analogs of the immunomodulatory, anti-angiogenic, and anti-neoplastic oral drug thalidomide. Similar to thalidomide, the primary target of both drugs is thought to be cereblon, the substrate-binding subunit of Cullin-RING E3 ubiquitin ligase 4 (CRL4^CRBN^), to promote the degradation of Ikaros lymphocyte differentiation factors [40,41,42]. The down-stream effects of lenalidomide and pomalidomide include inhibition of NF-κB [43] and the PI3K/mTor axis [44], as well as the oncogenic transcription factor and non-GCB marker IRF4 [45,46], probably indirectly as a result of the physical interaction with cereblon. Beyond the cytotoxic effect of lenalidomide and pomalidomide, the microenvironment modulating activity of both drugs includes proinflammatory repolarization of tumor-associated macrophages [47] and the activation of T cells and natural killer (NK) cells [41,42].

Lenalidomide alone or in combination with rituximab as a salvage therapy approach was investigated in a phase 1 study in 14 patients with recurrent or refractory PCNSL [48]. This phase 1 study enrolled 14 patients and determined that 15 mg daily of oral lenalidomide on 21 of 28 days was the maximum tolerated dose. The overall response rate was 68% and the median PFS was 6 months. Moreover, translational investigations suggested an association of relapse during lenalidomide therapy with cerebrospinal fluid activity of the immune tolerance-inducing enzyme indoleamine-2,3 dioxygenase [48,49]. 

In a phase 2 clinical trial of lenalidomide in combination with rituximab in 45 patients with recurrent or refractory PCNSL (*n* = 34) or primary intravitreal lymphoma (*n* = 11), 20–25 mg daily of oral lenalidomide was administered on 21 of 28 days with intravenous rituximab at 375 mg/m^2^ on day 1 for a total of 8 cycles, followed by maintenance lenalidomide at 10 mg daily. Grade 3/4 neutropenia occurred in 20 patients (44%), requiring a dose reduction in 19, and 15 patients (33%) experienced serious adverse events during the induction phase. The overall response rate among the 34 patients with PCNSL was 65% and the median PFS was 3.9 months [50]. Higher CD4/CD8 T cell ratios at baseline were associated with longer PFS, supporting the presumed immunomodulatory effect of lenalidomide. 

Several ongoing clinical trials explore the tolerability and efficacy of lenalidomide in combination with other standard and experimental treatments (Table 1), including three randomized phase 2 clinical trials in the first line setting comparing: (i) HD-MTX plus rituximab with versus without lenalidomide (NCT04481815), (ii) maintenance therapy with lenalidomide versus procarbazine following induction therapy with MTX plus rituximab and procarbazine in patients aged ≥ 70 years, and (iii) R-MPV (MTX plus rituximab, procarbazine and vincristine) plus ibrutinib versus lenalidomide (NCT04446962). Moreover, one randomized phase 2 trial explores the addition of ibrutinib versus lenalidomide in combination with MTX, rituximab, and etoposide in the recurrent or refractory setting (NCT04129710).

In a phase 1 study of pomalidomide in combination with dexamethasone in 29 patients with recurrent or refractory PCNSL, a maximum tolerated dose of 5 mg daily for 21 days every 28 days was determined, with the most common grade 3/4 toxicity being neutropenia in 21% of patients [51]. Among the 25 patients eligible for response assessment, the overall response rate was 48% and the median PFS was 5.3 months [51]. 

### 2.4. Other Molecularly Targeted Agents

Biological rationales exist for other small molecule inhibitors. Bcl-2 is expressed by the vast majority of PCNSL [52]. The Bcl-2 inhibitor venetoclax obtained approval for the treatment of chronic lymphatic leukemia [53]. An ongoing phase 1 study is exploring the feasibility of combined treatment with venetoclax and the anti-CD20 antibody obinutuzumab in patients with recurrent or refractory PCNSL (NCT04073147). The cerebral spinal fluid to plasma ratio of venetoclax in human subjects is in the range of 1:300 [54], indicating a poor crossing of the blood-brain barrier and suggesting that efficacy may depend on blood-brain barrier disruption. Proteasomal degradation of phosphorylated IkB is a prerequisite for activation of NF-κB, lending rationale for the brain-penetrating proteasome inhibitor marizomib [55]. The pre-clinical studies suggest that the silencing of the mutant *MYD88* gene expression can be achieved by utilizing histone deacetylase inhibitors and that this epigenetic reprogramming exerts synergy with ibrutinib in DLBCL [56,57]. It needs to be awaited which of these strategies will be investigated in the clinical setting.

## 3. Immune Checkpoint Inhibitors

### 3.1. Background

In recent years, drugs targeting the cytotoxic T lymphocyte-associated protein 4 (CTLA-4) or programmed cell death protein 1 (PD-1) pathways have also investigated in the context of primary and secondary brain tumors. Promising data were obtained when patients with brain metastasis were treated with immune checkpoint inhibitors [58], suggesting that the tumor localization in the brain does not preclude the therapeutic activity of these drugs. In contrast, PD-1 blockade has so far not conferred a survival benefit in patients with primary brain tumors such as glioblastoma [59]. The rather low expression of PD-1 and PD-L1 in gliomas, their rather low mutation load, and the immunosuppressive microenvironment might explain the lack of activity of checkpoint inhibitors. However, as lymphomas differ significantly from gliomas and other primary brain tumors, it has generally been considered a reasonable and promising strategy to test immune checkpoint inhibitors against PCNSL.

### 3.2. The Immune Signature of PCNSL

The presence of PD-L1 on tumor cells or PD-1 on tumor-infiltrating bystander cells may be an important prerequisite for clinical responses to PD-1 blockade [60]. Therefore, several studies looked at the expression of PD-1 or its ligands in tumor tissue of PCNSL patients with varying results. Comprehensive genetic analyses revealed copy gain and chromosomal translocation as key mechanisms of PD-L1 ligand upregulation. In a series of PCNSL, 67% had 9p24.1/PD-L1/PD-L2 copy gain and copy number-associated increased expression of the 2 ligands [61]. On the transcriptional level, high PD-1 and PD-L2 expression in PCNSL tissue specimens were associated with a poor prognosis [62]. On the protein level, tumor cells in PCNSL tissue specimens were rarely positive for PD-L1 (10%) or PD-1 (20%). However, 60% of tumor-infiltrating lymphocytes (TIL) were PD-1-positive. PD-L1-expressing tumor-associated macrophages were found in 20% of tumors. The authors did not find a correlation between tumor-infiltrating CD8+ T cells and PD-1 or PD-L1 expression [63]. Another study reported only 4% PD-L1-positive tumor cells in PCNSL tissue specimens, but 52% of the samples had PD-L1-positive cells in the tumor microenvironment. PD-L1 expression by tumor cells was associated with longer overall survival (*p* = 0.0177), whereas PD-L1 expression on cells in the microenvironment did not correlate with survival [64]. The expression of PD-L1 in macrophages and microglia cells infiltrating PCNSL was observed by other authors in a sub-fraction of tumors [65]. In a series of 48 PCNSL patients, high PD-L1 expression, defined as >5% staining, was found in 37.5% of tumor tissues, and intermediate expression (1–5% staining) was observed in 29.2%. PD-1 expression was found in 12 of the 14 examined tumors. No correlation between PD-1 and PD-L1 expression was observed [66]. Finally, in a series of 71 PCNSL tumor specimens, immunohistochemistry revealed PD-1 expression in 16 samples. PD-L1 was present in 42/71 tissues. The authors did not find a correlation between PD-1 or PD-L1 expression and gender, proliferation rate, or cell of origin [67]. In summary, most data point to a variable expression of PD-1 and its ligands in a fraction of PCNSL. 

Beyond tumor cell-expressed PD-L1, tumor mutational burden (TMB) was considered a potential predictive marker for response to PD-1 blockade [68]. In PCNSL, a TMB of ≥5 mutations per megabase (mt/Mb) was found in 41/42 tumors. Notably, 8 samples (19%) displayed high TMB (≥17 mt/Mb) and 71.4% of cases had intermediate TMB (7–16 mt/Mb). Microsatellite instability was not detected in any sample [66]. High tumor tissue PD-L1 expression was also associated with higher levels of soluble PD-L1 (sPD-L1) in the serum of PCNSL patients. Furthermore, sPD-L1 serum values in PCNSL patients were higher than in healthy control subjects. High sPD-L1 levels were associated with an increased risk for tumor recurrence, as well as shorter PFS and OS [69]. Further analyses are required to determine whether sPD-L1 may be used as a biomarker to identify patients who are most likely to derive benefit from PD-1 blockade.

### 3.3. Efficacy of PD-1 Blockade in Preclinical PCNSL Models

The therapeutic targeting of the PD-1 pathway was tested in a preclinical model of CNS lymphoma using A20 murine lymphoma cells, which express PD-L1 [70]. Immunocompetent mice were injected with A20 cells in the left periventricular area. Anti-PD-1 antibodies were administered intraperitoneally weekly starting at day 8 after tumor implantation. The anti-PD-1 treatment resulted in prolonged survival compared to untreated animals, with 50% of the mice still alive at day 70. The ex vivo analysis of the tumors by immunohistochemistry demonstrated a significant increase in tumor-infiltrating CD8+ T cells. In long-term surviving mice, no lymphoma cells were detectable, suggesting complete eradication of the tumor upon PD-1 therapy.

### 3.4. Clinical Data on Immune Checkpoint Inhibitors in PCNSL

Immune checkpoint inhibitors have not been systematically investigated in PCNSL thus far and the available evidence relies on small case series and anecdotal reports on the use of drugs targeting the PD-1 pathway. PD-1/PD-L1 inhibitors may be used as single agents or in combination with other treatment modalities. As expected, most data on the use of PD-1 inhibitors in PCNSL comes from patients with recurrent or relapsed tumors, as no standard of care is established for this situation [71]. Nevertheless, with a growing body of evidence on their use in PCNSL patients, the upfront use of PD-1 inhibitors or other immune checkpoint inhibitors may represent a novel therapeutic strategy, which might be of particular interest for patients who do not qualify for HD-MTX-based regimens.

In a series of four patients with relapsed or refractory PCNSL, and one patient with CNS relapse of primary testicular lymphoma (PTL), all patients responded to treatment with the PD-1 inhibitor nivolumab. Four of the patients achieved a complete response, while one had a partial response. Three of the patients were progression-free for more than 12 months [72]. The toxicity was mostly mild, including pruritus and fatigue. Renal insufficiency occurred in one patient and was considered unrelated to the nivolumab therapy. In general, there was a correlation between mutation load in the tumor tissue and response to immune checkpoint inhibition [68]. The tumor mutational burden might be a surrogate marker for the presence of neoantigens recognized by antigen-specific T cells, which become activated following PD-1 blockade. Terziev et al. identified a PCNSL patient with a tumor that exhibited high mutational burden [73]. In this particular patient, immunohistochemistry also revealed the presence of PD-1-positive tumor-infiltrating T cells. Due to these findings, a decision was made to treat the patient with autologous transplantation and subsequent nivolumab maintenance therapy, which resulted in a durable complete remission.

The combination of immune checkpoint inhibitors and other drugs may result in additional or even synergistic activity, but it also harbors the risk of increased toxicity. Ambady et al. reported on six patients with refractory or relapsed CNS lymphoma who were treated with a PD-1 inhibitor and rituximab. Of these six patients, three had systemic lymphoma with isolated CNS relapse and three had PCNSL that recurred following HD-MTX-based therapy. Five of the six patients had more than one line of prior therapy. A complete response was achieved in three patients (50%), which was durable in two of them. In one patient, progressive disease was diagnosed after the first dose, which led to additional treatment with WBRT and a subsequent partial response. As with other tumor entities, the imaging findings might have been misinterpreted as progressive disease but could have also represented immune checkpoint inhibitor-induced pseudoprogression [74]. A complete remission was also achieved in a PCNSL patient upon the administration of nivolumab in combination with a dendritic cell vaccination. Despite multiple lines of prior therapy, this immunotherapeutic concept resulted in a response for 10 months [75].

Graber et al. treated five patients, with primary or secondary CNS lymphoma refractory, to other therapies with the PD-1 inhibitor pembrolizumab. They observed durable, complete remissions in two patients, while the other three deteriorated after the first infusion [76]. In line with these findings, treatment with pembrolizumab resulted in a partial response in a patient with primary mediastinal B cell lymphoma and CNS involvement [77]. Although comparisons between primary and secondary CNS lymphomas are limited because of the different underlying biology, these data suggest that lymphoma manifestations in the CNS are sensitive to PD-1 inhibition. In summary, the available data support the further development of PD-1/PD-L1 inhibitors against B cell malignancies involving the CNS.

## 4. Conclusions

Therapeutic progress in the field of PCNSL has been limited in recent decades, with most efforts aimed at improving the efficacy of induction therapy and defining the role of consolidation treatment. Beyond radiation therapy and classical chemotherapeutic agents, only the CD20-targeting antibody rituximab was introduced in many treatment concepts despite the lack of conclusive data on its activity against CNS lymphomas. Novel therapeutic approaches with targeted agents and immunotherapeutic drugs may be particularly attractive in elderly or frail patients who are not eligible for protocols involving HD-MTX. Although comprehensive studies are lacking, these new treatment options may also have a more favorable toxicity profile, including less detrimental effects on cognitive function. The impact of novel drugs on neurocognition requires thorough analyses of large patient cohorts with adequate follow-up [78]. In summary, data from larger and ideally randomized trials are needed to determine if targeted agents (summarized in Table 1) or immune checkpoint inhibitors become available for clinical routine in the management of PCNSL patients. Prospective trials evaluating PD-1 inhibitors in PCNSL are ongoing. The CheckMate 647 study is a phase 2, open-label, single-arm study, which explores the safety and efficacy of nivolumab in patients with recurrent or relapsed PCNSL or PTL. The data of this trial will help in clarifying the role of PD-1 inhibitors in PCNSL and may lead to a subsequent randomized trial.

## Figures and Tables

**Table 1:** Ongoing clinical trials of targeted therapies in PCNSL.

Study	Setting	Phase	Identifier
*Randomized clinical trials*			
Rituximab plus MTX with versus without lenalidomide	First line	2	NCT04481815
Maintenance lenalidomide versus procarbazine following induction therapy with MTX plus rituximab and procarbazine	First line, age ≥ 70 years	2	NCT03495960
R-MPV (MTX, rituximab, procarbazine, and vincristin) plus ibrutinib versus lenalidomide	First line	2	NCT04446962
MRE (MTX, rituximab, and etoposide) plus ibrutinib versus lenalidomide	Recurrent or refractory	2	NCT04129710
*Uncontrolled studies*			
Acalabrutinib plus durvalumab	Recurrent or refractory	1	NCT04462328
Rituximab plus ibrutinib plus lenalidomide	Recurrent or refractory	1	NCT03703167
MTX and rituximab plus lenalidomide plus nivolumab	First line	1	NCT04609046
TEDD (temozolomide, etoposide, doxil, dexamethasone) plus intrathecal cytarabine plus ibrutinib	Recurrent or refractory	1	NCT02203526
Venetoclax plus obinutuzumab	Recurrent or refractory	1	NCT04073147
MTX and rituximab plus lenalidomide	First line	1/2	NCT04120350
MTX and rituximab plus ibrutinib	Recurrent or refractory	1/2	NCT02315326
Copanlisib plus ibrutinib	Recurrent or refractory	1/2	NCT03581942
Rituximab plus pemprolizumab plus ibrutinib	Recurrent or refractory	1/2	NCT04421560
Ibrutinib maintenance following induction with MTX and rituximab	First line, age 60–85	2	NCT02623010
Rituximab plus lenalidomide	First line	2	NCT04627753
MTX, rituximab and temozolomide plus lenalidomide	First line	2	NCT04737889
Nivolumab plus ibrutinib	Recurrent or refractory	2	NCT03770416
Orelabrutinib	Recurrent or refractory	2	NCT04438044
